# Comparative transcriptome analysis reveals deep molecular landscapes in stony coral *Montipora* clade

**DOI:** 10.3389/fgene.2023.1297483

**Published:** 2023-11-07

**Authors:** Tingyu Han, Xin Liao, Zhuojun Guo, J.-Y. Chen, Chunpeng He, Zuhong Lu

**Affiliations:** ^1^ State Key Laboratory of Bioelectronics, School of Biological Science and Medical Engineering, Southeast University, Nanjing, China; ^2^ Guangxi Key Lab of Mangrove Conservation and Utilization, Guangxi Mangrove Research Center, Beihai, China; ^3^ Nanjing Institute of Geology and Paleontology, Nanjing, China

**Keywords:** scleractinian coral, full-length transcriptome, genetic conservation, gene expression profile, biomineralization, immune response, stress response, adaptation

## Abstract

**Introduction:** Coral reefs, among the most invaluable ecosystems in the world, face escalating threats from climate change and anthropogenic activities. To decipher the genetic underpinnings of coral adaptation and resilience, we undertook comprehensive transcriptome profiling of two emblematic coral species, *Montipora foliosa* and *Montipora capricornis*, leveraging PacBio Iso-Seq technology. These species were strategically selected for their ecological significance and their taxonomic proximity within the Anthozoa class.

**Methods:** Our study encompassed the generation of pristine transcriptomes, followed by thorough functional annotation via diverse databases. Subsequently, we quantified transcript abundance and scrutinized gene expression patterns, revealing notable distinctions between the two species.

**Results:** Intriguingly, shared orthologous genes were identified across a spectrum of coral species, highlighting a substantial genetic conservation within scleractinian corals. Importantly, a subset of genes, integral to biomineralization processes, emerged as exclusive to scleractinian corals, shedding light on their intricate evolutionary history. Furthermore, we discerned pronounced upregulation of genes linked to immunity, stress response, and oxidative-reduction processes in *M. foliosa* relative to *M. capricornis*. These findings hint at the presence of more robust mechanisms in *M. foliosa* for maintaining internal equilibrium and effectively navigating external challenges, underpinning its potential ecological advantage. Beyond elucidating genetic adaptation in corals, our research underscores the urgency of preserving genetic diversity within coral populations.

**Discussion:** These insights hold promise for informed conservation strategies aimed at safeguarding these imperiled ecosystems, bearing ecological and economic significance. In synthesis, our study seamlessly integrates genomic inquiry with ecological relevance, bridging the gap between molecular insights and the imperative to conserve coral reefs in the face of mounting threats.

## 1 Introduction

As widely acknowledged, coral reefs, having undergone evolutionary processes spanning millions of years in our oceans, stand as one of the planet’s most invaluable ecosystems (Hoegh-Guldberg, 1999; van de Water et al., 2022). They provide refuge for approximately 30% of marine organisms (Smith, 1978; Copper, 1994; Reaka-Kudla, 1997; Spalding and Grenfell, 1997). However, the deleterious consequences of climate change, overfishing, pollution, and an array of other anthropogenic activities have wrought havoc upon coral reefs on a global scale (Carpenter et al., 2008; Wilkinson, 2008; Normile, 2009; Yu, 2012). A recent report by the nonprofit organization Coral Reef Alliance underscores the disconcerting fact that coral reefs continue to experience degradation. The repercussions of this degradation are profound, with nearly one trillion dollars annually at stake, encompassing aspects such as sustenance, tourism, and coastal defense. At present, 75% of these reefs are facing various threats, with predictions indicating that this alarming figure will increase to 90% by the year 2030. Consequently, an urgent call to action resounds for the preservation of coral reefs. While the challenges facing coral reefs are multifaceted and complex, the solution lies in our ability to delve deeper into the molecular intricacies of coral biology.

With the rapid advancement of biotechnology, multi-omics resources for coral research have significantly expanded, encompassing genomics (Shinzato et al., 2011; Voolstra et al., 2017; Shumaker et al., 2019; Shinzato et al., 2021), transcriptomics (Takeuchi et al., 2016; Zhou et al., 2017; Zhang et al., 2018), proteomics (Drake et al., 2013; Takeuchi et al., 2016; Drake et al., 2020), metagenomics (Gust et al., 2014; Wood-Charlson et al., 2015; Zhang et al., 2017; Sun et al., 2020), and single-cell transcriptomics (Hu et al., 2020; Levy et al., 2021; Steger et al., 2022; Hu et al., 2023). These developments offer invaluable assets for elucidating the concealed responses of corals to environmental stressors, decoding the genetic foundations of their resilience, and comprehending the intricacies of their symbiotic associations. However, it is essential to note that the focus of these investigations remains limited relative to the vast diversity of coral species, numbering in the thousands. This limitation is especially notable in the case of *Montipora* corals, where sequencing data resources are particularly limited (Hauck, 2007; Wang et al., 2018; Helmkampf et al., 2019; Shumaker et al., 2019; Cho et al., 2020; Williams et al., 2021). *Montipora*, one of the most widespread genera of reef-building corals in the Indo-Pacific, is the second most species-rich coral genus globally, only surpassed in species numbers by *Acropora*, another member of the same family (Veron, 1984; Veron, 2000). *Montipora* corals are renowned for their diverse growth forms, including encrusting, laminar, and branching morphologies, which can be quite distinct from other Acroporid corals. This diversity in growth forms is believed to be a result of their unique adaptations to different environmental conditions (Veron, 1984; Veron, 2000). Moreover, some *Montipora* species have exhibited a remarkable level of resilience to environmental stressors such as high sea surface temperatures (Williams et al., 2021; Drury et al., 2022a; Drury et al., 2022b; Henley et al., 2022) and ocean acidification (Kavousi et al., 2016; Sekizawa et al., 2017). Researchers have shown great interest in their ability to withstand and recover from bleaching events, making them a focal point in the study of coral health and adaptation. Furthermore, studies have unveiled the high genetic diversity found within populations of *Montipora* species (Takahashi-Kariyazono et al., 2015; Drury et al., 2022a; Caruso et al., 2022). This genetic diversity may play a crucial role in their adaptability to changing environmental conditions. Expanding the multi-omics resources dedicated to *Montipora* corals represents a critical step towards addressing these knowledge gaps. This comprehensive approach aims not only to bridge the existing research deficit but also to provide a holistic understanding of the underlying mechanisms and adaptations that govern coral species’ responses to environmental fluctuations. Such insights have the potential to significantly advance coral conservation efforts and deepen our scientific comprehension of coral ecosystems.

Generally, genomes are better suited for researching long-term evolutionary processes and understanding the genetic basis of species divergence due to their stability and comprehensive nature (Ng and Kirkness, 2010). Conversely, transcriptomes offer particular value in the investigation of gene expression patterns, responses to environmental fluctuations, and short-term adaptations. However, a practical challenge emerges concerning the relatively high sequencing costs associated with genomes. Additionally, the quality of genome assemblies for some available coral species may be less than optimal, possibly due to inherent complexities. Hope arises with the introduction of full-length transcriptome sequencing technologies, such as PacBio Iso-Seq protocol. This approach allows for the direct sequencing of transcripts up to 10 kb in length, eliminating the need for a reference genome (Rhoads and Au, 2015). Consequently, it becomes feasible to employ this strategy in exploring the evolutionary relationship among multiple species lacking reference genomes.

In this study, we employed the Iso-Seq strategy to generate comprehensive full-length transcriptome maps for two coral species within the genus *Montipora*, namely, *M. foliosa* and *M. capricornis*. These closely related species share a recent common ancestor, resulting in a high degree of gene conservation. Over time, subtle genetic differences have emerged since their divergence from this common ancestor, and these differences are likely to be relevant to their unique adaptations. Furthermore, the presence of similar environmental pressures in their respective habitats has led to parallel genetic changes, providing robust evidence of adaptation. Despite these similarities, *M. foliosa* and *M. capricornis* exhibit differences in growth forms and environmental preferences that suggest they may experience varying selection pressures and environmental challenges. *M. foliosa* is known for its encrusting growth form (Dai, 2009) and is commonly found in shallow reef environments (Montaggioni and Faure, 1997; Montaggioni and Martin-Garin, 2020). In contrast, *M. capricornis* displays a range of growth forms, including flat plates in tiers or whorls, columns, encrusting growth, and irregularly contorted laminae (Veron et al., 2016) and is typically found in deeper and calmer reef environments (Veron, 2000). By studying these two species, we aim to explore how differences in the genetic underpinnings of their respective adaptations and responses to environmental stressors may manifest in their transcriptomes. To achieve this, we utilized a short-read sequencing approach for transcriptome correction and quantitative gene expression analysis. Using this dataset, we identified and analyzed orthologous genes among multiple cnidarians and subsequently constructed a phylogenetic tree to elucidate their evolutionary relationships. Following this, we delved into an exploration of the environmental selection pressures impacting these two *Montipora* coral species. Finally, we carried out a differential gene expression analysis to discern the disparities in gene expression profiles between these two species. This research contributes to our broader understanding of coral biology by unraveling the genetic and transcriptomic distinctions between closely related coral species. It equips us with essential knowledge for the conservation and restoration of these fragile yet ecologically vital ecosystems, shedding light on the mechanisms of coral adaptation and resilience in the face of environmental challenges.

## 2 Materials and methods

### 2.1 Sample collection and laboratory culture

Two specimens of coral colonies, *M. foliosa* and *M. capricornis* (Supplementary Figure S1), were collected from the vicinity of the Xisha Islands (15°40′–17°10′N, 111°–113°E) at water depths ranging from approximately 5–10 m. These coral colonies were carefully transported to a controlled laboratory aquarium environment and subjected to a 1-month acclimation period. Subsequently, each coral colony was meticulously fragmented into three distinct fragments, serving as biological replicates. These fragmented coral specimens underwent an additional acclimation period of 2 months within the laboratory aquarium before being utilized for the extraction of mRNA.

All coral samples were initially cultured in a standardized RedSea tank (redsea575, Red Sea Aquatics Ltd., London, United Kingdom) following the established Berlin Method. The tank was maintained at a constant temperature of 26°C, with a salinity level of 1.025. Specific equipment used in the controlled environment included three coral lamps (AI^®^, Red Sea Aquatics Ltd., London, United Kingdom), a high-performance protein skimmer (Reef Octopus Regal 250S, Honya Co., Ltd., Shenzhen, China), a precision water chiller (tk1000, TECO Ltd., Port Louis, Mauritius), two wave devices (VorTech™ MP40, EcoTech Marine Ltd., Bethlehem, PA, United States), and a calcium reactor (Reef Octopus OCTO CalReact 200, Honya Co., Ltd., Shenzhen, China).

### 2.2 mRNA extraction and sequencing

To ensure the availability of sufficient high-quality mRNA (>15 µg) for constructing a PacBio cDNA library and three Illumina cDNA libraries, we meticulously processed three biological replicate samples from each coral, which were obtained in the previous step. This process involved manually grinding the samples into a fine powder, utilizing a mortar and pestle that remained continuously frozen in liquid nitrogen to preserve the integrity of the samples throughout the mRNA extraction process. All mRNA extraction procedures strictly adhered to the manufacturer’s instructions. Total RNA was isolated using TRIzol^®^ LS Reagent (Thermo Fisher Scientific, 10296028, Waltham, MA, United States) and subsequently treated with DNase I (Thermo Fisher Scientific, 18068015, Waltham, MA, United States). High-quality mRNA was isolated using a FastTrack MAG Maxi mRNA Isolation Kit (Thermo Fisher Scientific, K1580-02, Waltham, MA, United States). The mRNA extraction procedure followed these steps: 1) grinded coral samples (ensuring the samples remained submerged in liquid nitrogen at all times); 2) added TRIzol^®^ LS reagent at a ratio of approximately 1:3 (sample to reagent) to the ground samples; 3) allowed the samples to thaw naturally; 4) continued adding TRIzol^®^ LS reagent until the samples were completely dissolved, and then dispensed them into 50 mL centrifuge tubes; 5) centrifuged at 4°C and 3,000 rpm for 5–15 min; 6) collected the supernatant into 50 mL centrifuge tubes; 7) added BCP (Molecular Research Center, BP 151, Cincinnati, OH, United States) to the above centrifuge tubes in a 5:1 ratio of sample to reagent, shook well, and allowed it to stand for 10 min; 8) centrifuged at 4°C and 10,500 rpm for 15 min; 9) obtained the supernatant, added an equal volume of Isopropanol (Amresco, 0918-500ML, Radnor, PA, United States), mixed well, and incubated overnight at −20°C; 10) centrifuged at 4°C and 10,500 rpm for 30 min, discarded the supernatant; and 11) rinsed them twice with 75% Ice Ethyl alcohol, Pure (Sigma-Aldrich, E7023-500ML, Taufkirchen, München, Germany). Finally, three samples from each coral were extracted in equal amounts (totaling >10 µg) and combined for full-length transcriptome sequencing utilizing the PacBio Sequel II platform, while the remaining portions (>1.5 µg per sample) were utilized for short-read sequencing by Illumina HiSeq X Ten platform.

Before establishing the library, the quality of the total RNA was rigorously assessed through a series of key quality control measures. RNA degradation and contamination were scrutinized by electrophoresis on 1% agarose gels. RNA purity, determined by the OD260/280 ratio, was checked using the NanoPhotometer^®^ spectrophotometer (IMPLEN, Westlake Village, CA, United States). Quantification of RNA concentration was performed with the Qubit^®^ RNA Assay Kit on a Qubit^®^ 2.0 Fluorometer (Thermo Fisher Scientific, Waltham, MA, United States), and RNA integrity was evaluated using the RNA Nano 6000 Assay Kit on the Agilent Bioanalyzer 2100 system (Agilent Technologies, Santa Clara, CA, United States). These comprehensive assessments ensured that the RNA used for library preparation was of high quality and suitable for downstream applications.

### 2.3 Raw data processing procedure

A total of 1.5 µg of mRNA per sample served as the input material for RNA sample preparations. Sequencing libraries, generating 6 Gb sequencing data per sample, were constructed using the NEBNext^®^ Ultra™ RNA Library Prep Kit (E7530L) for Illumina^®^ (NEB, Ipswich, MA, United States), following the manufacturer’s guidelines. Index codes were introduced to attribute sequences to each respective sample.

In summary, the mRNA was initially purified from total RNA using poly-T oligo-attached magnetic beads. Fragmentation occurred with the assistance of divalent cations under elevated temperature conditions in NEBNext First-Strand Synthesis Reaction Buffer (5×). The first-strand cDNA was synthesized using a random hexamer primer and M-MuLV Reverse Transcriptase (RNase H−). Subsequently, second-strand cDNA synthesis was carried out using DNA Polymerase I and RNase H, with any remaining overhangs converted into blunt ends through exonuclease/polymerase activities. After adenylation of the 3′ ends of DNA fragments, an NEBNext Adaptor with a hairpin loop structure was ligated to prepare for hybridization. To selectively target cDNA fragments within the 250–300 bp range, library fragments underwent purification using an AMPure XP system (Beckman Coulter, Beverly, Brea, CA, United States). Then, 3 µL of USER Enzyme (NEB, Ipswich, MA, United States) was applied to size-selected, adaptor-ligated cDNA at 37°C for 15 min, followed by 5 min at 95°C prior to PCR. PCR was performed with Phusion High-Fidelity DNA polymerase, Universal PCR primers, and Index (X) Primer. Finally, PCR products were purified using the AMPure XP system, and library quality was assessed using the Agilent Bioanalyzer 2100 system. Clustering of the index-coded samples occurred on a cBot Cluster Generation System, employing the TruSeq PE Cluster Kit v3-cBot-HS (Illumina, San Diego, CA, United States), following the manufacturer’s instructions. After cluster generation, the library preparations were sequenced on an Illumina HiSeq X Ten platform, yielding paired-end reads.

The Iso-Seq library (20 Gb sequencing data) was meticulously prepared following the isoform sequencing protocol, utilizing the Clontech SMARTer^®^ PCR cDNA Synthesis Kit (Clontech Laboratories, now Takara Laboratories, 634926, Mountain View, CA, United States) in conjunction with the BluePippin Size Selection System protocol, as outlined by Pacific Biosciences (PN 100-092-800-03). In brief, Oligo(dT)-enriched mRNA underwent reverse transcription to produce cDNA through the SMARTer PCR cDNA Synthesis Kit. The synthesized cDNA was subsequently amplified through polymerase chain reaction (PCR) using the BluePippin Size-Selection System protocol. The Iso-Seq library was constructed, involving full-length cDNA damage repair, terminal repair, and the attachment of SMRT dumbbell adapters. The sequences of the unattached adapters at both ends of the cDNA were eliminated through exonuclease digestion. The resulting cDNA was combined with primers and DNA polymerase to form a complete SMRT bell library. Upon qualification of the library, the PacBio Sequel II platform was employed for sequencing, aligning with the library’s effective concentration and data output requirements.

The initial processing of Illumina sequencing raw reads in fastq format involved the utilization of in-house Perl scripts. During this step, we extracted clean data by filtering out reads containing adapters, reads with poly-N sequences, and low-quality reads. Additionally, we calculated essential quality metrics such as Q20, Q30, GC-content, and assessed the level of sequence duplication for the clean data. Subsequently, all subsequent analyses were conducted exclusively on this high-quality, clean data.

To process the PacBio sequencing raw data, we used SMRTlink v8.0 software (Pacbio, Menlo Park, CA, United States) to generate high-quality consensus sequences. The circular consensus sequence (CCS) was generated from subread BAM files with the following parameters: min_length 50, min_passes 1, max_length 15,000. CCS.BAM files were output, which were then classified into full-length and non-full-length reads using lima, removing polyA using refine. Full-length fasta files produced were then fed into the cluster step, which performed isoform-level hierarchical clustering [n×log(n)], followed by final arrow polishing, hq_quiver_min_accuracy 0.99, bin_by_primer false, bin_size_kb 1, qv_trim_5p 100, and qv_trim_3p 30. These sequences were subsequently subjected to correction for any additional nucleotide errors using LoRDEC v0.7 software (Salmela and Rivals, 2014). To enhance data clarity, redundancies were eliminated from the dataset using CD-HIT v4.6.8 software (parameters: −c 0.95 −T 6 −G 0 −aL 0.00 −aS 0.99) (Fu et al., 2012), resulting in a set of unique sequences referred to as unigenes. These unigenes were then aligned against reference genomes of the Symbiodiniaceae family using GMAP v2017-06-20 software (Wu and Watanabe, 2005). Sequences that successfully mapped to the Symbiodiniaceae reference genomes were classified as Symbiodiniaceae sequences, while the remaining sequences were categorized as coral sequences. This categorization allowed for the subsequent analysis of these sequences, ensuring a more focused examination of relevant data.

### 2.4 Gene function annotation, ORF prediction, and expression quantification

Gene functions were extensively annotated using various databases and software tools, including NT (NCBI non-redundant nucleotide sequences) analyzed with BLAST 2.7.1+ software (Altschul et al., 1990), NR (NCBI non-redundant protein sequences) (Li et al., 2002), KOG/COG database (Clusters of Orthologous Groups of proteins) (Tatusov et al., 2003), Swiss-Prot (A manually annotated and reviewed protein sequence database) (Bairoch and Apweiler, 2000), and KEGG (Kyoto Encyclopedia of Genes and Genomes) (Kanehisa et al., 2004) assessed with Diamond v0.8.36 BLASTX software (Buchfink et al., 2015). Pfam (Protein family) (Finn et al., 2016) database analysis was conducted using the HMMER 3.1 package (Mistry et al., 2013). Additionally, GO (Gene Ontology) terms (Ashburner et al., 2000) were integrated into the comprehensive annotation process. The relevant figures illustrating these annotations were created using Origin 2022 software (OriginLab Corporation, Northampton, MA, United States).

The ANGEL v2.4 software (Shimizu et al., 2006) was employed for open reading frame (ORF) prediction, with the longest ORF selected as the coding sequence (CDS). Short-read sequencing data was then mapped to the full-length transcriptome reference using kallisto v 0.50.0 software with the parameter -k 31, while keeping the other parameters at their default settings (Bray et al., 2016). Subsequently, gene expression profiles, including read count and TPM (Transcripts Per Million), were obtained for each sample. The figures depicting relevant analyses, such as Figure 2, were generated using R package ggplot2 v3.4.3 and pheatmap v1.0.12 with R v4.3.1.

### 2.5 Phylogenetic analysis and Ka/Ks ratio estimation

Protein sequences from eight cnidarian species were analyzed using OrthoFinder v2.5.4 (Emms and Kelly, 2015; Emms and Kelly, 2019) and Diamond v0.9.24 (Buchfink et al., 2021) with the parameter -S diamond -M msa to predict orthogroups, resulting in the identification of 427 single-copy OGs. Sequences within the same OG were aligned using Muscle v5.1 (Edgar, 2022), and divergent or ambiguously aligned blocks were removed from the protein sequence alignments using Gblocks v0.91b with the parameter -b4 = 5 -b5 = h -t = p -e = .2 (Talavera and Castresana, 2007). Subsequently, all sequences from the same species were concatenated using SeqKit v2.5.1 (using the commands seqkit sort and seqkit seq -w 0) (Shen et al., 2016).

Prior to constructing the phylogenetic tree, ProtTest v3.4.2, using the command “prottest-3.4.2.jar -i all.phy -all-distributions -F -AIC -BIC -tc 0.5 -threads 28 -o prottest.out” (Guindon and Gascuel, 2003; Darriba et al., 2011) was employed to predict and select an appropriate amino acid substitution model. Finally, a maximum likelihood analysis was conducted on the concatenated sequences, which totaled 160,318 amino acids in length, using RAxML v8.2.12 using the command “standard-RAxML-master/raxmlHPC-PTHREADS-SSE3 -T 28 -f a -x 123 -p 123 -N 1000 -m PROTGAMMAIJTTF -k -O -o Edia, Nvec \-n all.tre -s all.fa” (Stamatakis, 2014) with 1,000 bootstraps. TimeTree 5 (Kumar et al., 2022) was used to estimate the divergence time between the two species, and then the MCMCtree function in the PAML program package v4.10.6 (Yang, 1997; Yang, 2007) was utilized to estimate the divergence time of the phylogenetic tree. The results were subsequently analyzed using Tracer v1.7.2 (Rambaut et al., 2018), where ESS values were checked. Generally, an ESS value exceeding 200 suggests that the phylogenetic tree has likely converged.

Additionally, the protein sequences of the two *Montipora* species were aligned using NCBI’s BLAST + v2.13.0 with the parameter -evalue 1e-5 -max_target_seqs 1 -num_threads 8 -outfmt 6 to obtain reciprocal best hits, and subsequently, the non-synonymous (Ka) and synonymous (Ks) substitution rates (Ka/Ks ratio) were calculated using ParaAT v2.0 (Zhang et al., 2012), clustalw2 v2.1 (Larkin et al., 2007), and KaKs_Calculator2.0 (Wang et al., 2010) using the command “ParaAT.pl -h XX.homolog -n XX.cds -a XX.pep -m clustalw2 -p proc -f axt -g -k -o paml” with the default model averaging (MA) approach.

### 2.6 Differential gene expression analysis and GO enrichment

To enable the comparison of gene expression profiles across different species, we adopted a method outlined in a previously published article (Brawand et al., 2011). Here’s the step-by-step process. Initially, we filtered genes within each sample based on their expression values within the inner quartile range. Next, from the previously mentioned reciprocal best hits between the two *Montipora* species, we selected gene pairs with e-values less than 1e-08 and sorted them in descending order of pident (percentage of identical matches). We chose the top 100 orthologous gene pairs in this manner. We then took the intersection of the results obtained in the previous two steps for each sample, resulting in a set of conserved orthologous genes unique to each sample. We calculated the median expression value for these conserved orthologous genes within each sample. By normalizing these median values across all samples, we obtained scaling factors. These factors were subsequently used to scale the gene expression profiles for all samples, making them comparable.

Additionally, we calculated *p*-values using the SCBN v1.18.0 R package (Zhou et al., 2019). Genes with both scbn_p_value and median_p_value below 1e-06 were considered DEGs. Specifically, genes with an absolute log2 (fold change) greater than 2 were identified as significant DEGs and selected for further analysis.

The construction of PCA plot was based on the TPM expression values of orthologous genes in various samples from the two species. We utilized the R packages ggplot2 v3.4.3, factoextra v1.0.7, and FactoMineR v2.8 (Le et al., 2008) for this task. The generation of volcano plot also employed the ggplot2 v3.4.3 package, while heatmap creation utilized pheatmap v1.0.12. Furthermore, we conducted GO enrichment analyses separately for upregulated and downregulated significant DEGs using clusterProfiler v4.8.2 (Wu et al., 2021) and enrichplot v1.20.1. To construct PPI networks, we employed the STRING v12.0 database (https://string-db.org/) and visualized the networks using Cytoscape v3.10.1 (https://cytoscape.org/).

## 3 Results

### 3.1 Full-length transcriptome profiling and functional annotation strategies

**FIGURE 1:**
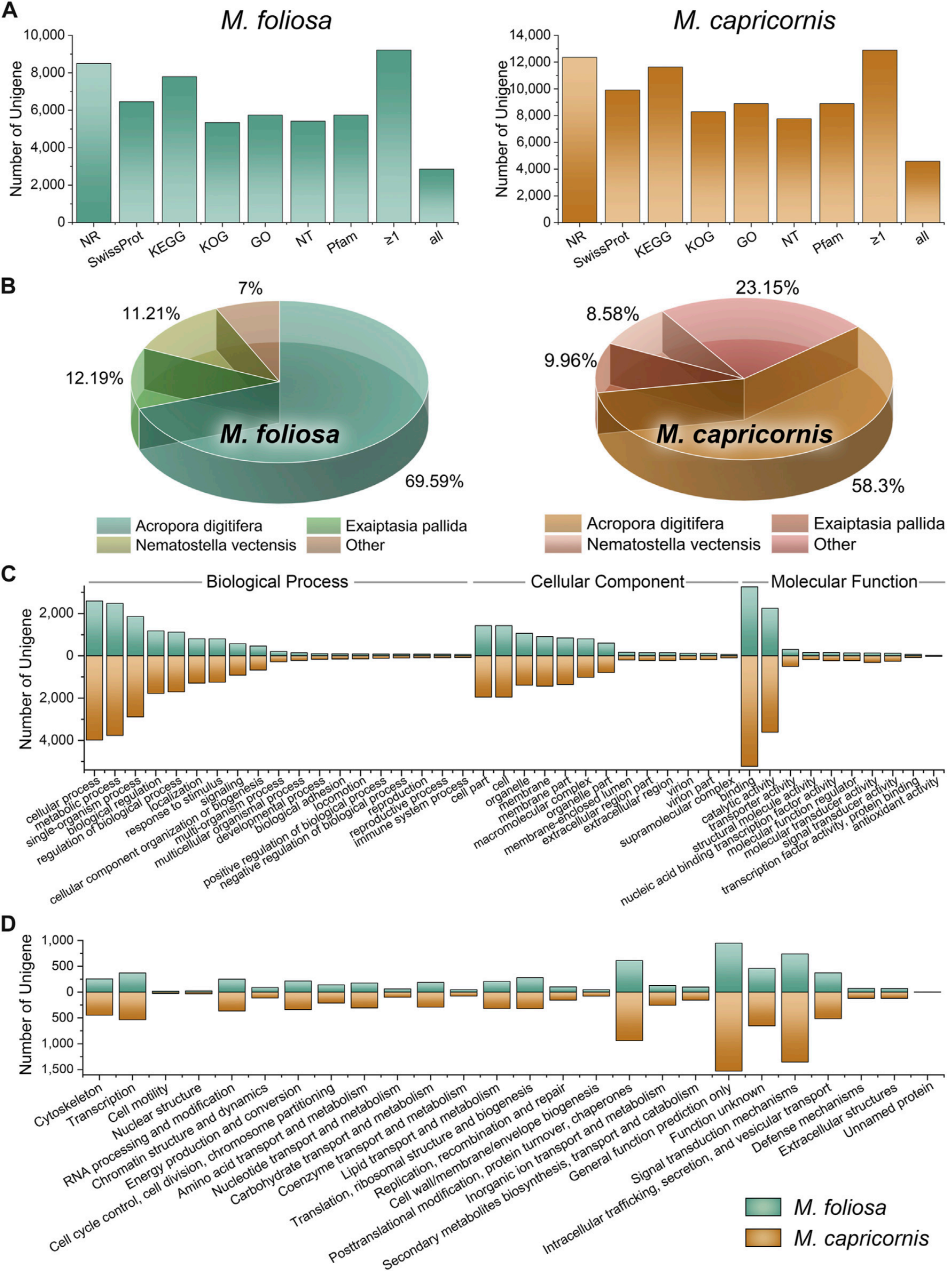
Function annotation of unigenes in two coral species. **(A)** Functional annotation results in the multiple functional databases. The horizontal axis represents the different databases, and the vertical axis represents the number of unigenes annotated in different databases, with a minimum of one database (≥1), and all databases. **(B)** The top three species with the greatest number of sequence hits for two coral species. **(C)** GO annotation classifications. **(D)** KOG annotation classifications.

In this study, we utilized the PacBio Iso-Seq method to comprehensively profile the full-length transcriptomes of two distinct coral species, *M. foliosa* and *M. capricornis*, resulting in substantial raw data (21.44 Gb for *M. foliosa* and 23.00 Gb for *M. capricornis*). To ensure data quality, rigorous filtering, error correction, and redundancy reduction processes were conducted, yielding 10,905 high-quality unigenes for *M. foliosa* and 13,857 for *M. capricornis* (Table 1). To attain a comprehensive understanding of gene functionalities, we performed gene functional annotation on the unigene sequences using various databases, including NR, NT, Pfam, KOG, Swiss-Prot, KEGG, and GO (Figure 1A; Supplementary Table S1).

**TABLE 1:** PacBio Iso-Seq transcriptome analysis for *M. foliosa* and *M. capricornis*.

Sample name	*M. foliosa*	*M. capricornis*
Polymerase reads (Gb)	21.44	23.00
Subreads (Gb)	20.24	22.06
Polished consensus (Number)	26,455	30,879
Unigenes (Number)	10,905	13,857
Mean length (bp)	1,472	1,992
Minimum length (bp)	69	121
Maximum length (bp)	5,942	7,925
N50 length (bp)	1,678	2,125
N90 length (bp)	905	1,365

Intriguingly, within the *M. foliosa* transcriptome, an impressive 84.44% of unigenes were annotated to at least one functional database, with 26.15% being annotated across all databases tested (77.95% in NR, 59.20% in Swiss-Prot, 71.50% in KEGG, 48.99% in KOG, 52.65% in GO, 49.64% in NT, and 52.65% in Pfam). The proportions were notably higher in *M. capricornis*, with 92.98% of unigenes annotated to at least one database, and 33.07% being annotated across the entire functional spectrum (89.15% in NR, 71.48% in Swiss-Prot, 83.94% in KEGG, 59.80% in KOG, 59.80% in GO, 59.80% in NT, and 64.16% in Pfam).

A particularly robust outcome emerged from the NR annotation, where the top three species consistently represented within the results were classified as members of the Anthozoa class (Figure 1B). Impressively, these Anthozoan species accounted for a substantial proportion of the annotated sequences—93% and 76.85% in *M. foliosa* and *M. capricornis*, respectively. This remarkable concordance underscores the reliability of the annotation outcomes. The uniform presence of Anthozoa-related annotations in both transcriptomes enhances the confidence in the functional assignments, validating their congruence with the biological essence of the coral specimens and their close taxonomic association with the Anthozoa group. These collective findings solidify the credibility and robustness of the annotation protocol.

Notably, the striking agreement observed in the GO and KOG classification distributions across both coral transcriptomes underscores a fundamental alignment in their functional and evolutionary attributes (Figures 1C, D). This congruence is suggestive of a convergence in the underlying molecular processes and biological functions, indicative of conserved functional attributes intrinsic to these coral species. The parallel distribution patterns within both GO and KOG classifications highlight shared molecular functions, biological processes, and cellular components. This coherence offers substantial validation for the reliability of functional annotations, amplifying the likelihood of shared functional adaptations, potentially driven by analogous ecological niches or environmental challenges.

### 3.2 Quantifying transcript abundance and expression patterns in coral transcriptomes

**FIGURE 2:**
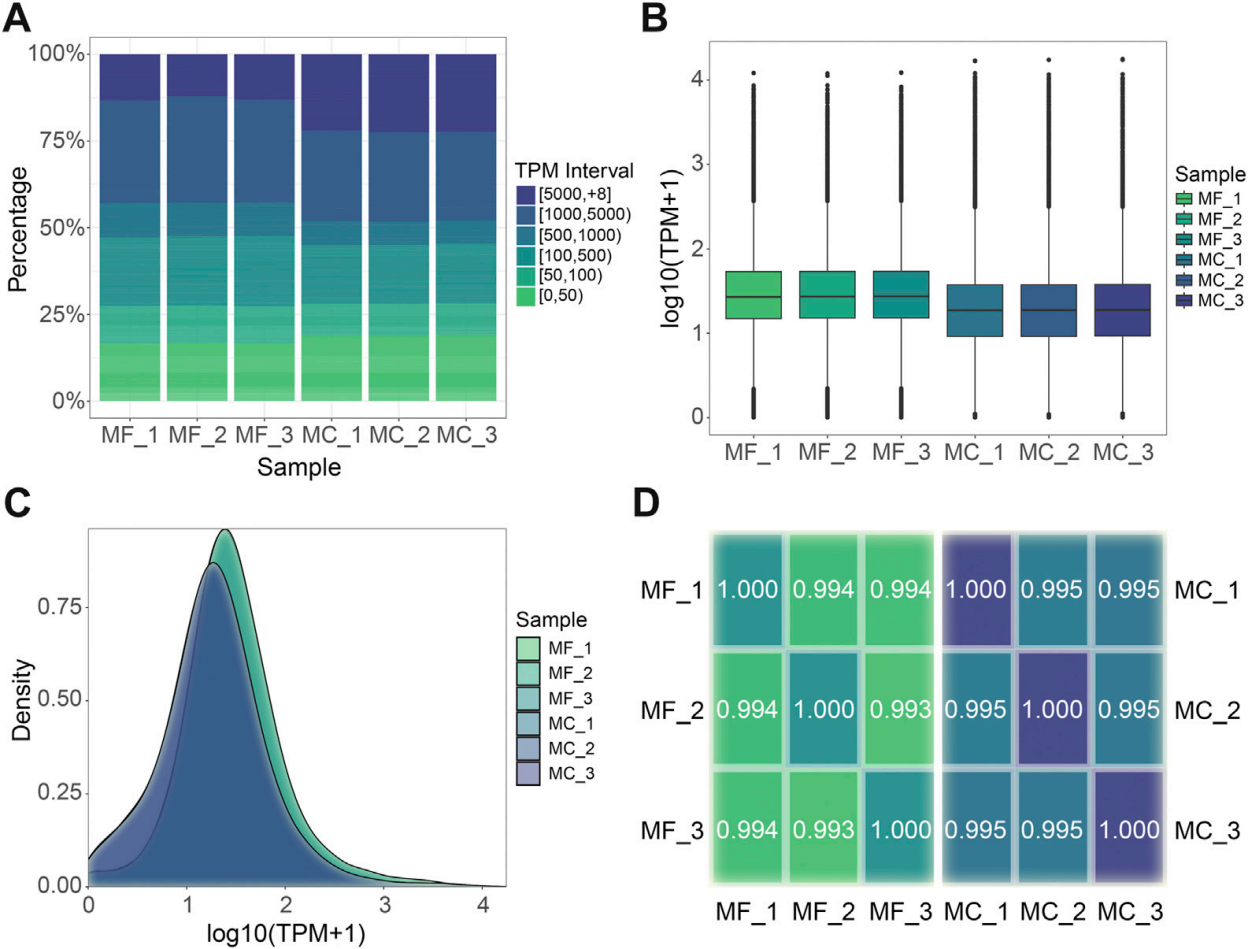
Gene expression level analysis. **(A)** TPM interval distribution in different samples. **(B)** TPM box plot. Each box plot displays five statistical values, including the maximum, upper quartile, median, lower quartile, and minimum, arranged in a top-to-bottom order. **(C)** TPM density distribution. **(D)** Spearman’s rank correlation among samples in each species respectively. The closer the value is to 1, the better the correlation. MF: *M. foliosa* and MC: *M. capricornis*.

Utilizing the distinct full-length transcriptomes of *M. foliosa* and *M. capricornis* as individualized reference sequences, we executed a targeted alignment procedure for the sequencing data derived from their respective sets of three biological replicate samples. The resulting outcomes are presented in Table 2. Subsequently, we proceeded to ascertain and quantify the expression values of individual unigenes, as detailed in Supplementary Table S2. Upon examining the distribution of transcript abundance across varying TPM intervals, it becomes evident that within *M. capricornis*, a notable increase in the proportion of both highly and lowly expressed unigenes is observed when contrasted with *M. foliosa* (Figure 2A). Additionally, a more intricate analytical exploration through the use of boxplot (Figure 2B) and density distribution plot (Figure 2C) provides a visually informative perspective, further substantiating the observation that *M. foliosa* displays a more concentrated distribution of unigene expression levels compared to *M. capricornis*. In moving forward, we conducted Spearman’s rank correlation coefficient (rs) to meticulously evaluate the interrelationships amongst samples within each coral species (Figure 2D). Notably, the computed rs values consistently surpass the threshold of 0.99, not only emphasizing the remarkably high congruence in expression patterns among samples but also underscoring the rationality of our biological replicate sampling strategy, thus substantiating its suitability for subsequent analysis processes.

**TABLE 2:** Transcriptome mapping assessment for two coral species.

Sample name	*M. foliosa*			*M. capricornis*		
Biological replicates	MF_1	MF_2	MF_3	MC_1	MC_2	MC_3
Ref Unigenes (No.)	10,905			13,857		
Total Reads (No.)	32,236,782	26,161,661	22,505,803	32,128,553	28,411,018	41,970,484
Total mapped Reads (No.)	18,079,927	14,616,989	12,629,696	17,860,605	15,817,694	23,400,162
Total mapped Reads (%)	56.1	55.9	56.1	55.6	55.7	55.8
Uniquely mapped Reads (No.)	15,131,228	12,252,962	10,564,576	14,800,746	13,096,911	19,374,380
Uniquely mapped Reads (%)	46.9	46.8	46.9	46.1	46.1	46.2

### 3.3 Orthologous gene analysis reveals genetic conservation and adaptation in coral taxa

In addition to the two focal coral species targeted in this investigation, our study leveraged publicly available genome datasets encompassing *M. capitata* (Shumaker et al., 2019) from the same genus, along with *A. digitifera* (Shinzato et al., 2011) and *A.millepora* (Fuller et al., 2020), both of which, notably, belong to the Acroporidae family, as well as *Stylophora pistillata* (Voolstra et al., 2017) from the Pocilloporidae family. Furthermore, we incorporated two anemone species, *Nematostella vectensis* (Fletcher et al., 2023) and *Exaiptasia diaphana* (Baumgarten et al., 2015), for the purpose of orthologous group (OG) clustering (Supplementary Table S3.1). By conducting an in-depth analysis of protein sequences across these diverse taxa, we identified a total of 27,814 distinct OGs across the Cnidaria, with 25,247 OGs present in Scleractinia, 23,934 in Acroporidae (complex corals), and 21,636 in *Montipora* (Supplementary Tables S3.2, S3.3).

**FIGURE 3:**
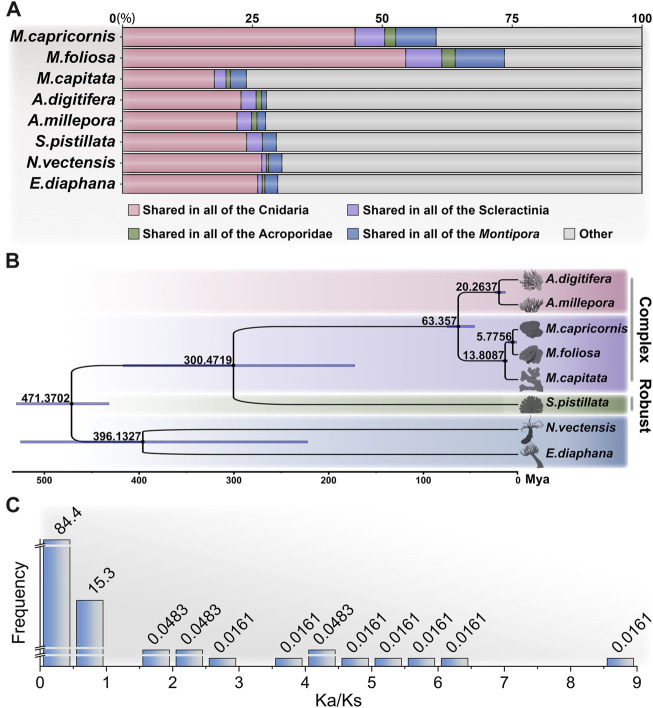
Analysis of orthologous groups and phylogenetic relationships of cnidarians. **(A)** Proportions of shared orthologous group genes among different groups: Cnidaria, Scleractinia, Acroporidae, and *Montipora*. **(B)** Phylogenetic analysis of cnidarians showing the divergence times, constructed using the JTT + I + G + F model with 1,000 bootstrap. **(C)** Analysis of environmental pressure selection in two *Montipora* species.

Of particular significance, a subset of 4,134 OGs was found to be shared universally among the scleractinian coral species (Figure 3A), encompassing approximately half of the total unigenes detected in the two focal coral species of our investigation (55.06% in *M. foliosa* and 49.14% in *M. capricornis*). This outcome underscores a notable degree of genetic congruence evident among this taxonomically diverse set of organisms. The identified shared OGs collectively emphasize a substantial level of genetic conservation within scleractinian corals, reflecting an underlying theme of shared biological attributes across these species. The prominent convergence of shared OGs between the two studied coral species and the broader group of scleractinians imparts an intriguing implication: an intrinsic genetic foundation that likely contributes to their shared ecological roles and adaptations. This observed genetic similarity further attests to a high degree of evolutionary persistence in fundamental genetic elements. The conserved genes highlighted in this overlap likely govern pivotal biological processes that are integral to the vital functions of both coral species. Crucially, the significant overlap of orthologous genes between the two examined coral species (71.37% in *M. foliosa* and 61.73% in *M. capricornis*) underscores a marked genetic affinity between these species (Supplementary Table S3.2). This shared genetic underpinning signifies a pronounced level of evolutionary preservation in fundamental genetic components, alluding to conserved genetic traits governing essential biological processes shared by both coral species.

In addition, a total of 225 OGs were found to be exclusive to scleractinian corals, devoid of any corresponding presence within the anemone species (Supplementary Table S3.4). Notably, approximately half of these distinctive OGs lack explicit annotations, potentially representing genes intrinsic to stony corals, albeit their functional elucidation remains pending. Surprisingly, an intriguing discovery pertains to a collection of previously reported genes associated with biomineralization processes, including hemicentin-2, skeletal aspartic acid-rich protein 1, α-collagen, ZP domain-containing protein, calmodulin, major yolk protein, uncharacterized skeletal organic matrix protein (USOMP), and aspartic and glutamic acid-rich protein (Zoccola et al., 2004; Drake et al., 2013; Mummadisetti et al., 2021). This assortment implies a plausible indispensability of these genes in the orchestration of coral skeletal formation. Concurrently, the inventory comprises 744 OGs present within anemone species, yet conspicuously absent within scleractinians, suggesting potential scenarios whereby these specific OGs underwent loss within the stony coral clade or emerged post-divergence.

The application of phylogenomic analysis on 427 single-copy OGs yielded robust phylogenetic relationships among the studied species (Supplementary Figure S2; Supplementary Table S3.5). Subsequently, utilizing available fossil-based divergence times as references, we inferred the divergence times of distinct taxa based on protein sequence (Figure 3B). To ensure the precision of constructing the divergence time tree, we heightened the iteration count of the algorithm to secure the convergence of results, specifically requiring an effective sample size (ESS) greater than 500 (Supplementary Table S3.6). The outcomes were consistent with prior investigations, revealing a closer phylogenetic relationship between complex corals (including genus *Montipora* and *Acropora*), followed by robust corals, while sea anemones exhibited a notably more distant relation (Shumaker et al., 2019; Shinzato et al., 2021). Regarding the results, among all the species scrutinized, the target corals, *M. foliosa* and *M. capricornis*, exhibited the closest phylogenetic relationship. Consequently, to gain deeper insights into their evolutionary dynamics, we are poised to delve into the analysis of selective pressure based on orthologous genes, thereby undertaking an assessment of the variation in evolutionary rates within these two coral species.

A total of 10,656 reciprocal best-hit sequence pairs were obtained within the two focal coral species. Subsequently, we estimated the Ka/Ks ratio for each gene pair (Supplementary Table S3.7). It is noteworthy that certain gene pairs exhibited an exceptionally low Ks value, indicative of a lack of synonymous changes within these sequences. This observation implies minimal or negligible substitutions (NA) within the aligned gene sequences (Mo et al., 2020). Consequently, meticulously scrutinize gene pairs by implementing criteria such as *p*-value < 0.01 (calculated through Fisher’s exact test) and Ks > 0.001. This stringent filtering yielded a refined collection of 6,212 gene pairs (Figure 3C; Supplementary Table S3.8). Remarkably, among these pairs, approximately 99.74% displayed Ka/Ks ratios below 1, a pattern congruent with the expectations because of negative (purifying) selection commonly observed in many protein-coding regions (Nielsen, 2005). Conversely, only 16 pairs of orthologous genes indicated positive (adaptive) selection (Ka/Ks >> 1). Upon further annotation scrutiny, merely 4 of these pairs revealed explicit functional annotations, including neuronal pentraxin, neuroplastin (2 pairs), and cnidarian carbohydrate-associated protein (cnidCAP). CnidCAP shares homology with mannose-binding lectin-associated serine proteases (MASP), a component in the lectin-mediated activation of the classic complement pathway of innate immunity. Previous evidences have demonstrated a lectin-mediated interaction between the host and symbiont (Jimbo et al., 2000; Lin et al., 2000; Wood-Charlson et al., 2006). Furthermore, study has indicated that cnidCAP’s expression is suppressed during coral bleaching processes, implying a role in symbiosis maintenance. This observation implies that this mutualistic association might have originated from pathogenic influences and continues to be modulated by components of innate immunity (Hauck, 2007). Therefore, the positive selection observed in cnidCAP potentially bestows evolutionary benefits upon corals. Notably, prior research has already highlighted the upregulation of neuronal pentraxin-like genes in corals after 5 weeks of thermal stress (Williams et al., 2021). Additionally, neuroplastin, a glycoprotein belonging to the immunoglobulin superfamily of cell adhesion molecules (Owczarek and Berezin, 2012), also showed positive selection. This collective evidence indicates that these genes might have undergone adaptive evolution, possibly linked to environmental adaptation or functional alterations.

### 3.4 Cross-species differential gene expression analysis

**FIGURE 4:**
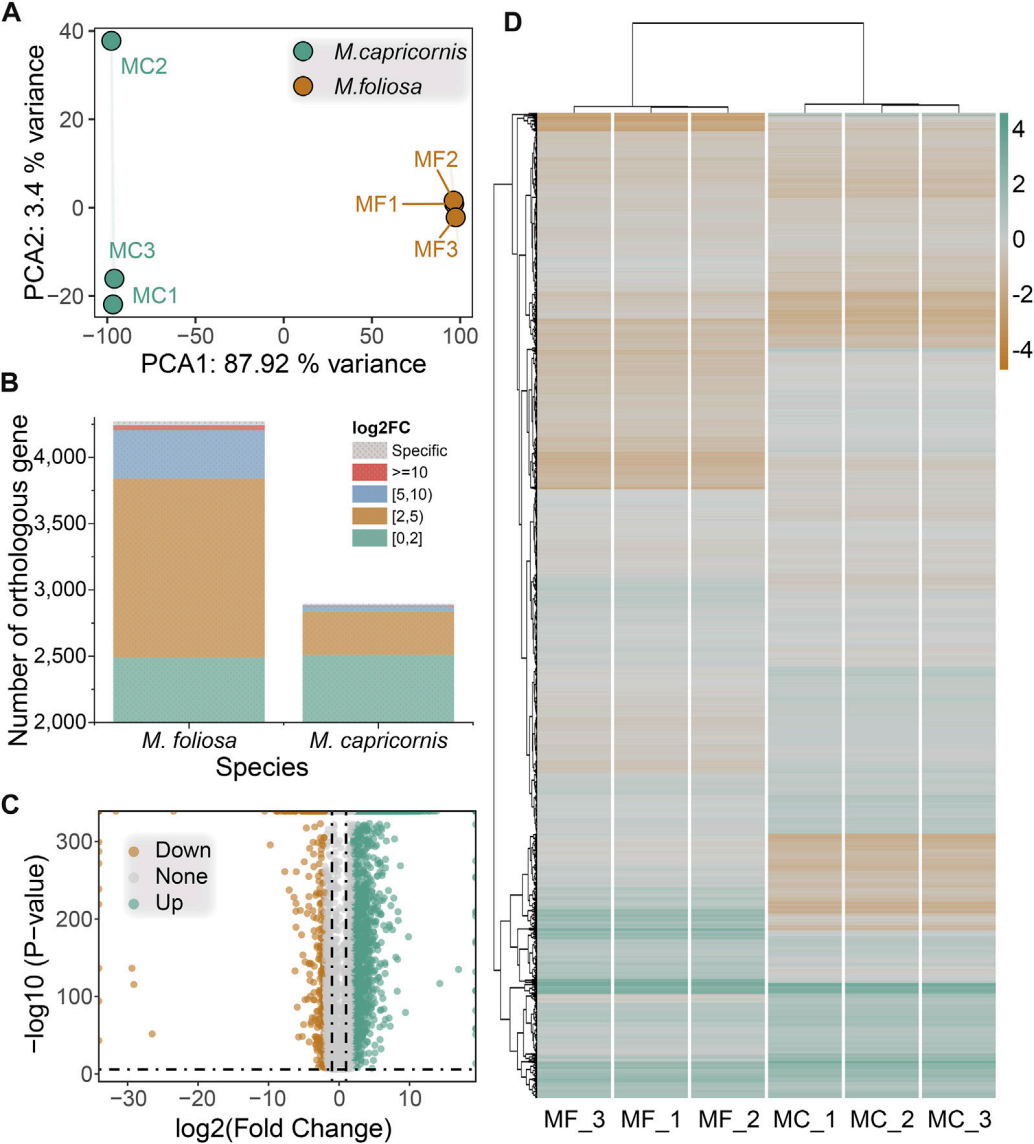
Cross-species differential gene expression analysis. **(A)** PCA of the biological replicate samples for the two coral species. **(B)** The distribution of the number of significantly differentially expressed genes in two coral species. **(C)** Volcano map of differentially expressed genes. **(D)** Heatmap of differentially expressed genes in biological replicate samples for the two coral species. Green for highly expressed genes, brown for lowly expressed genes.

In order to enable a meaningful comparative analysis of the transcriptomes between *M. foliosa* and *M. capricornis*, we adopted a normalization approach inspired by the methodology outlined in the work by Brawand et al. (2011) (Brawand et al., 2011). This normalization procedure was applied uniformly to the expression data from all samples, ensuring a consistent foundation for analysis (Supplementary Table S4). Leveraging the identified orthologous gene pairs, we performed principal component analysis (PCA) on the six samples. The PCA results revealed a slight separation of one *M. capricornis* sample from the rest, indicating a nuanced divergence. Nonetheless, when considered holistically, the samples from each respective coral species exhibited clustering tendencies (Figure 4A). This analysis enhances the comparability and coherent interpretation of the gene expression profiles across the distinct coral species.

Subsequent to the differential gene expression analysis, a total of 2,166 significantly differentially expressed orthologous gene pairs were identified. Among these, 1,782 exhibited upregulation in *M. foliosa*, including 30 that were uniquely expressed in this species. Conversely, 384 displayed upregulation in *M. capricornis*, encompassing 13 genes exclusive to this species (Figure 4B), a notably lower count than observed in *M. foliosa*. However, the distribution pattern of orthologous genes with differential expression across various expression ranges remained similar in both coral species, as indicated by the representation of log2(fold change) values. The majority of these genes are concentrated within the range of 2–5, accounting for 75.81% and 84.38% of these genes in *M. foliosa* and *M. capricornis*, respectively (Figure 4C). The next prevalent range is 5–10, constituting 20.48% and 10.68% in *M. foliosa* and *M. capricornis*, respectively. Only a limited number of significantly differentially expressed orthologous gene pairs, 36 and 6, respectively, displayed log2(fold change) values greater than or equal to 10. Additionally, for a visual representation of the variability in gene expression levels across multiple samples, we constructed a heatmap (Figure 4D). The results illuminated that the gene expression patterns among the three biological replicates of each coral species were remarkably consistent. In contrast, notable distinctions in gene expression patterns emerged between the two coral species, highlighting potential distinctions in their underlying regulatory mechanisms.

### 3.5 Functional analysis of differentially expressed orthologous genes

**FIGURE 5:**
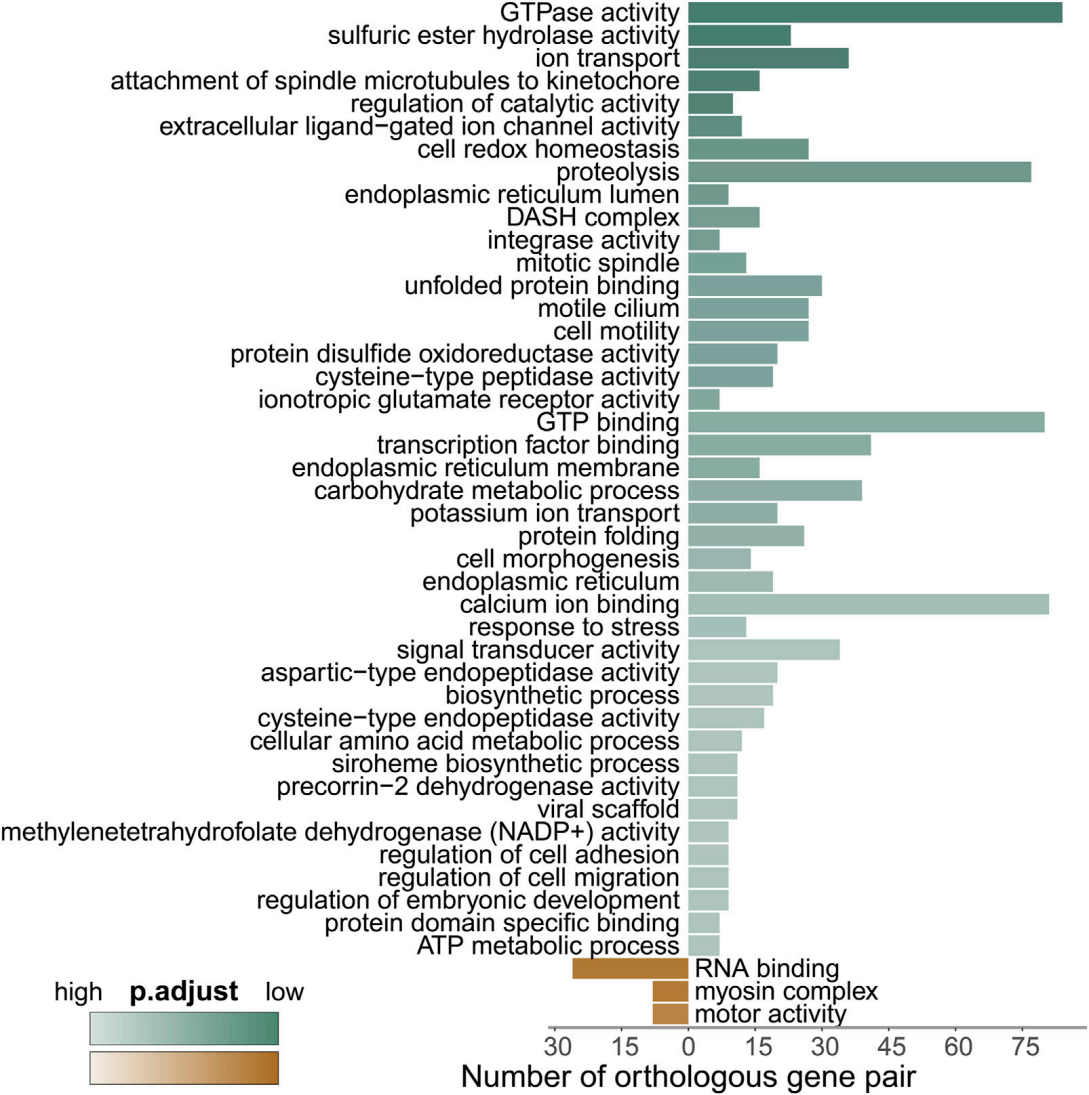
GO enrichment analysis of the differentially expressed genes. Green represents enriched GO terms for genes upregulated in *M. foliosa*, while brown represents enriched GO terms for genes upregulated in *M. capricornis*. The darker the color, the smaller the p.adjust value, indicating a higher level of enrichment.

To gain deeper insights, we conducted a GO enrichment analysis of the differentially expressed genes (DEGs) between the two coral species, as depicted in Figure 5 and Supplementary Table S5. Notably, compared to *M. capricornis*, *M. foliosa* exhibited a significant enrichment of upregulated genes in a total of 45 GO terms. Among these, the highest number of genes were enriched in terms associated with cellular processes involving the interaction and regulation of GTP molecules within cells. These enriched terms included GO:0003924 and GO:0005525. These DEGs primarily comprised members of critical protein families, such as the RAS superfamily proteins, tubulin superfamily proteins, and ATP-related proteins (Supplementary Figure S3A), indicating a significant presence of genes related to GTP-mediated cellular processes in *M. foliosa*.

Intriguingly, the analysis of DEGs associated with integrase activity (GO:0008907) uncovered a group of proteins closely linked to innate immunity, featuring leucine-rich repeat-containing protein 74A (LRRC74A) and NLRC3 (Supplementary Figure S3B). Further exploration also revealed the upregulation of leucine-rich repeat-containing protein 72 (LRRC72), while NLRC3-like gene expression was notably downregulated, indicating its higher expression in *M. capricornis* (Supplementary Figure S3B). Mammalian NLRC3 serves as a well-established negative regulator within the NOD-like receptor (NLR) family, primarily implicated in virus sensing processes (Zhang et al., 2014; Uchimura et al., 2018; Li et al., 2019). However, reports in fish suggest that NLRC3-like genes may exert both positive and negative regulatory functions in response to various types of pathogen infections (Fang et al., 2020; Chang et al., 2021). Moreover, teleost fish studies have noted an expansion of NLRC3, contributing to an increased abundance of NLRC3-like genes (Chang et al., 2021). These findings highlight the complexity of NLRC3 and NLRC3-like genes’ roles in the immune response, which may vary across different species and contexts.

**FIGURE 6:**
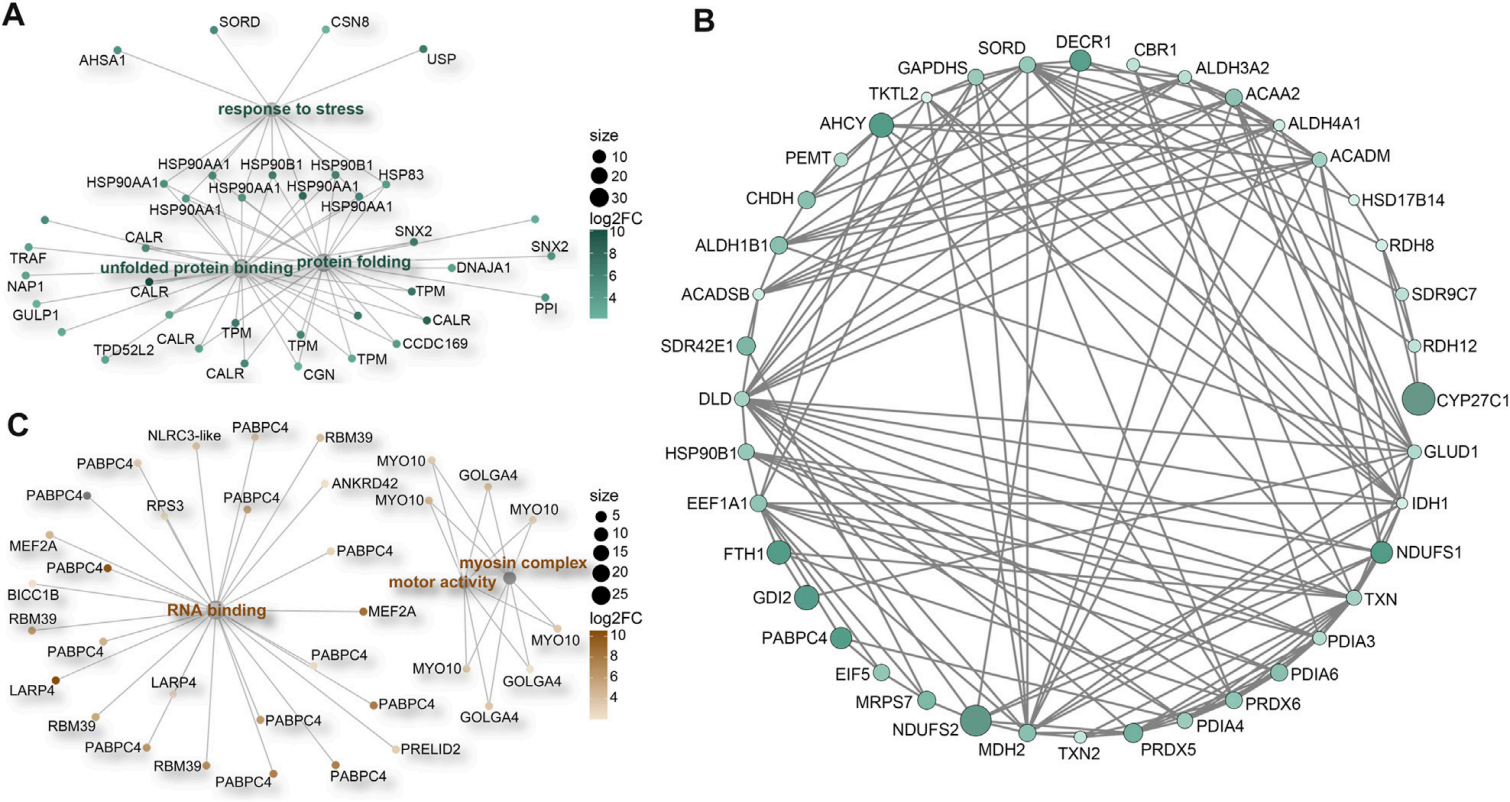
Protein-protein interaction (PPI) networks. **(A)** Enrichment of significant upregulated DEGs in stress response-related GO terms in *M. foliosa*. **(B)** PPI network among the significant upregulated DEGs related to oxidative-reduction processes in *M. foliosa*. **(C)** Enrichment of significant upregulated DEGs in GO terms in *M. capricornis*.

Our analysis additionally unveiled significant enrichment of upregulated genes in various GO terms related to stress responses (Supplementary Figure S3C). These encompassed terms like “unfolded protein binding” (GO:0051082), “protein folding” (GO:0006457), and “response to stress” (GO:0006950). Key participants within these processes included members of the HSP90 protein family, calreticulin (CALR) proteins, and tropomyosins (TPMs) (Figure 6A; Supplementary Figure S4). Furthermore, a distinct cluster of genes tied to oxidative-reduction processes was identified (Figure 6B; Supplementary Figure S5A). Notably, this cluster featured cytochrome P450 superfamily members (CYP27C1 and CYP46A1), previously reported to be upregulated in corals under heat stress conditions (Voolstra et al., 2009). Interestingly, these genes were exclusively detected in *M. foliosa*. Genes encoding NADH dehydrogenase (ubiquinone) iron-sulfur proteins (NDUFS1 and NDUFS2), rab GDP dissociation inhibitor beta (GDI2), adenosylhomocysteinase (AHCY), and soma ferritin exhibited significantly higher expression levels in *M. foliosa* compared to *M. capricornis*, with log2(fold change) values of approximately 10, emphasizing their role in species differentiation. Several genes in the peroxiredoxin family, particularly PRDX5 and PRDX6, were significantly upregulated in *M. foliosa*, while PRDX2 displayed significant upregulation in *M. capricornis*. Protein disulfide isomerases (PDIs), which play a catalytic role in protein folding, along with thioredoxins (TXN and TXN2) and thioredoxin reductases (TXNRDs), responsible for catalyzing the reduction of thioredoxin, were all significantly upregulated in *M. foliosa*.

In contrast, *M. capricornis* displayed a distinct profile, with upregulated DEGs significantly enriched in three specific GO terms (Figure 6C; Supplementary Figures S5B, S6). Core members of these terms included polyadenylate-binding protein 4 (PABPC4), RNA-binding protein 39 (RBM39), unconventional myosin-X (MYO10), and golgin subfamily A member 4 (GOLGA4). However, it is worth noting that the actual expression levels of these genes in both coral species were not notably high. Additionally, a set of genes related to biomineralization processes was found to be significantly upregulated exclusively in *M. foliosa*. This set comprised genes such as secreted acidic protein 1B/2, carbonic anhydrase 12/2, galaxin, USOMP5/3/7, and skeletal aspartic acid-rich protein 1 (Supplementary Figure S5C).

Finally, we examined genes with Ka/Ks ratios markedly greater than 1 and identified four gene pairs with significantly different expression profiles between the two coral species (Supplementary Figure S5D). Among these, three were upregulated (OG62, OG1162, and OG1526), although their functions remain unknown. One downregulated gene, referred to as cnidCAP (OG10550), which was previously mentioned in relation to potential involvement in innate immunity, was also observed. These findings collectively highlight the distinct gene expression patterns and biological processes contributing to the observed differences between *M. foliosa* and *M. capricornis*, particularly in the context of stress responses and oxidative-reduction processes.

## 4 Discussion

In this study, we conducted a comprehensive analysis of the full-length transcriptomes of two distinct coral species, *M. foliosa* and *M. capricornis*, to gain insights into their genetic conservation and adaptation mechanisms. Our findings shed light on several crucial aspects of these coral species, including gene functionalities, expression patterns, genetic conservation, and adaptation strategies.

Phylogenetic analysis of cnidarians revealed that among the three *Montipora* species, *M. foliosa* and *M. capricornis* are more closely related to each other, while they are more distantly related to *M. capitata*. This observed pattern may be attributed to their distinct morphologies, with the former two exhibiting a laminar growth form, while the latter displays an arborescent growth form. This phenomenon finds support in previous research, as indicated by a study involving a phylogenetic tree of 15 *Acropora* species (Shinzato et al., 2021). Within this tree, it was observed that species within each clade shared similar morphologies, including arborescent, hispidose, corymbose, table-shaped, and others. This discovery implies that there is a strong correlation between genetic divergence and the growth forms of coral species. Specifically, the consistent morphological characteristics within each phylogenetic clade suggest that the evolution of specific growth forms has occurred independently multiple times. These variations in growth forms may represent adaptations to particular ecological niches, environmental conditions, or selective pressures. Overall, this highlights the considerable plasticity of coral species in response to their surroundings and underscores the intricate relationship between morphology and evolutionary history in corals.

Here, we identified cnidCAP, a protein with a Ka/Ks ratio exceeding 1, along with a significant difference in expression levels between *M. foliosa* and *M. capricornis* [log2(fold change) = −3.75]. Importantly, despite this difference, both species displayed high expression levels of cnidCAP. This finding hints at a pivotal role for cnidCAP in coral evolution, potentially shaped by persistent positive selective pressure. Considering its association with the symbiotic relationship between corals and Symbiodiniaceae algae, as suggested by Hauck (2007), we propose several hypotheses targeting these corals: differing functional demands may exist, which potentially involving symbiotic regulation, pathogen defense, or responses to distinct environmental stressors; subtle adaptive distinctions influenced by their symbiotic partners or specific ecological contexts may exist; and ecological niches or lifestyles may be slightly divergent, potentially linked to symbiotic relationships, dietary preferences, or responses to environmental challenges. Further research could delve into the precise roles of cnidCAP in coral symbiosis and ecological adaptations, offering deeper insights into its biological significance.

The identification of a group of orthologous genes related to biomineralization presents a fascinating conundrum. These genes appear exclusively in scleractinian corals, yet traces of genes annotated to similar proteins are discovered in sea anemones. Takeuchi et al. (2016) unearthed striking similarities between skeletal organic matrix proteins (SOMPs) in the scleractinian coral *A. digitifera* and those found in sea anemones (Takeuchi et al., 2016). This uncovers the intriguing possibility that these proteins were inherited from a common ancestor, predating the divergence of corals and sea anemones. The existence of orthologous genes related to biomineralization in both groups suggests a shared genetic legacy, hinting at the presence of the calcification toolkit in their ancestral lineage. However, the subsequent evolution of these genes in corals resulted in the development of coral-specific SOMPs, integral to the calcification process. Sea anemones, although retaining some of these genes, appear to have diverged in function, lacking the calcification prowess of their coral counterparts. This revelation prompts profound questions regarding the evolutionary path that bestowed scleractinian corals with their calcification capabilities. The hypotheses proposed by Takeuchi et al., including the emergence of novel proteins through domain shuffling and rapid molecular evolution, offer glimpses into the potential mechanisms driving biomineralization’s evolution in corals. In essence, while orthologous genes reveal a shared genetic heritage, the intricate dance of subsequent adaptations in corals underscores the complexity of calcification’s evolution. Further investigations into the functional disparities and evolutionary narratives of these genes promise deeper insights into the enigmatic journey of biomineralization in corals, set apart from their sea anemone relatives.

Focusing on the target corals, *M. foliosa* and *M. capricornis*, we observed significant differences in the expression levels of certain biomineralization-related genes. Specifically, these genes displayed significant upregulation in *M. foliosa*, while such pronounced changes were not observed in *M. capricornis*. These genes, with the exception of carbonic anhydrase, fall under the category of SOMPs. Carbonic anhydrase, which catalyzes the conversion of metabolic CO_2_ into bicarbonate ions (HCO_3_^−^) both intracellularly and extracellularly under favorable pH conditions (Venn et al., 2009; Bertucci et al., 2013; Hopkinson et al., 2015), plays a crucial role in the biomineralization process. Within SOMPs, there are two subcategories: acid-rich proteins and binders, each fulfilling distinct roles in the biomineralization process. The acid-rich proteins include secreted acidic protein and skeletal aspartic acid-rich protein. These proteins are characterized by their high content of acidic amino acids and possess the capability to directly interact with amorphous calcium carbonate (ACC). This interaction promotes crystal nucleation, determines growth axes, and exerts control over crystal growth (Puverel et al., 2005; Mass et al., 2013). The prevalence of these acid-rich proteins in *M. foliosa* suggests their potential contribution to a more efficient biomineralization process in this coral species. Conversely, the binder proteins consist of galaxin and USOMPs, which play a role in orchestrating the assembly and arrangement of mineralization crystals. These proteins likely contribute to maintaining the structural integrity of the coral skeleton (Bhattacharya et al., 2016). Essentially, these observations provide insights into the potential mechanisms underlying the differences in biomineralization processes between *M. foliosa* and *M. capricornis*, with a focus on the differential expression of genes related to this process.

Furthermore, we have identified a significant upregulation of numerous genes related to innate immune (LRRC74A, LRRC72, and NLRC3), stress response (HSP90s, CALRs, and TPMs), and oxidative-reduction (CYP450s, NDUFSs, GDI2, AHCY, soma ferritin, PRDXs, PDIs, TXNs, and TXNRDs) processes in *M. foliosa* when compared to *M. capricornis*. These biological processes are intricately intertwined and play pivotal roles in maintaining an organism’s internal equilibrium and defending against external threats (Thorpe et al., 2004; Eberl, 2016). This heightened and coordinated upregulation of genes in *M. foliosa* suggests that it may have evolved to possess a superior capacity for maintaining internal stability and responding effectively to external challenges compared to *M. capricornis*. These processes are fundamental for the coral’s survival and its ability to adapt to its specific environmental conditions. The specific genes and pathways involved in these processes provide valuable insights into the potential mechanisms underlying the distinct ecological niches and environmental challenges faced by these two coral species. Further research is imperative to gain a comprehensive understanding of how these genetic disparities translate into functional advantages for *M. foliosa* and contribute to its ecological success. Another possibility is that the high expression of these genes in *M. foliosa* were already “frontloaded,” meaning they are maintained in an upregulated state under natural growth conditions. Research has demonstrated that an organism’s environmental history can influence its response to elevated temperatures and its overall tolerance to extreme events. Pre-exposure to stress has been shown to result in the upregulation of key genes involved in stress responses (Middlebrook et al., 2008; Middlebrook et al., 2010; Barshis et al., 2013; Krueger et al., 2017).

One limitation of this study is that it primarily relied on transcriptomic data and computational analyses, which provide valuable insights into gene expression patterns but require further experimental validation to confirm the functional roles of identified genes. Additionally, the study focused on a comparative analysis of two coral species, *M. foliosa* and *M. capricornis*, within specific environmental contexts. To gain a more comprehensive understanding of coral adaptation, future research should incorporate functional assays, long-term field studies, and a broader range of coral species, along with their genetic diversity. This would enable a deeper exploration of the ecological and evolutionary mechanisms that underlie coral resilience and adaptation in the face of ongoing environmental challenges.

## 5 Conclusion

In conclusion, this study has revealed significant differences in gene expression patterns and functional attributes between the coral species *M. foliosa* and *M. capricornis*. These differences suggest that *M. foliosa* may have evolved with a superior capacity for stress response, immunity, and biomineralization compared to *M. capricornis*. The shared genetic foundation observed in both species, particularly in orthologous genes, highlights a remarkable genetic conservation within scleractinian corals. However, the subtle but distinct variations in gene expression and functional attributes emphasize their ability to adapt to specific ecological niches and environmental challenges. Understanding the genetic basis of coral adaptation is crucial for coral conservation and the preservation of coral reef ecosystems. It provides insights into how corals might respond to ongoing environmental changes. Further research, including functional validation and field studies, is needed to unravel the precise mechanisms underlying these genetic disparities and their ecological implications. Ultimately, this knowledge can guide conservation efforts aimed at protecting these vital and vulnerable marine ecosystems.

## Data Availability

The data generated and analyzed in this study have been made openly accessible to the scientific community. The raw PacBio sequencing data can be retrieved from the Sequence Read Archive (SRA) at https://www.ncbi.nlm.nih.gov/sra (accessed on 6 September 2023) under the reference numbers SRX9416080 and SRX9416081, corresponding to *Montipora foliosa* and *Montipora capricornis*, respectively. The reference numbers for the corresponding short-read sequencing raw data are SRX9411986-8 for *M. foliosa* and SRX9411989, SRX9411932-3 for *M. capricornis*. Additionally, the processed data can be accessed on Figshare at https://figshare.com/s/90b155904e419f442d85 (accessed on 6 September 2023). These openly accessible resources serve to facilitate further investigations and foster collaborations in the field of coral research.
