# Supplementary material for: Comparative transcriptome analysis reveals deep molecular landscapes in stony coral Montipora clade
Source: Front Genet. 2023 Nov 7;14:1297483. doi: 10.3389/fgene.2023.1297483 (PMC10662330; doi:10.3389/fgene.2023.1297483)
Supplement: Supplementary file 6 [file Image1.pdf]

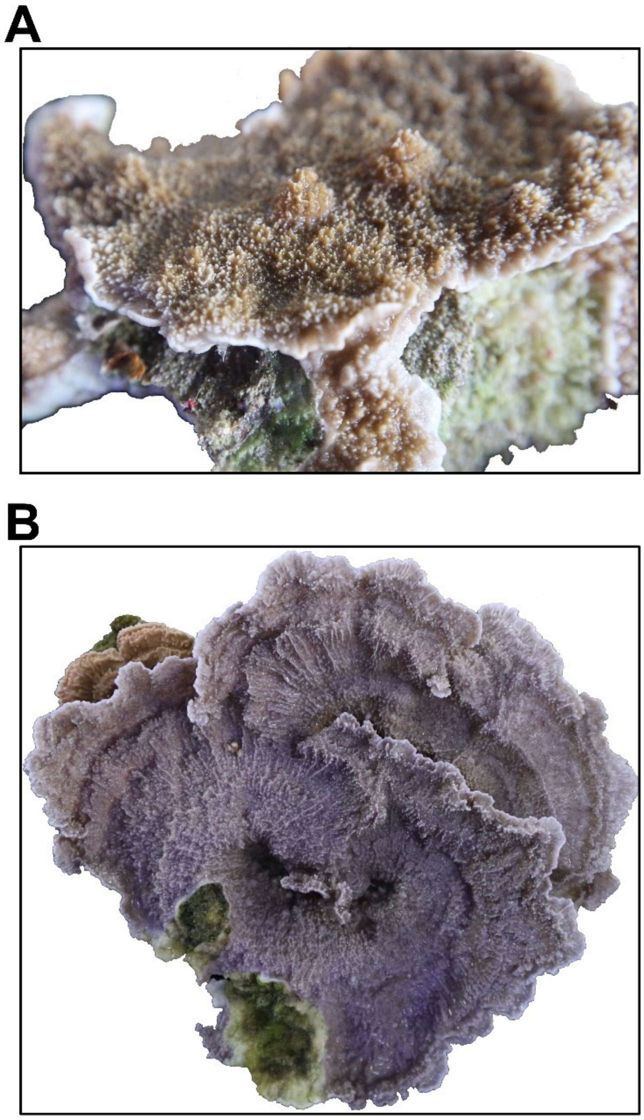

Supplementary Figure 1. High-resolution images of representative coral specimens. (**A**) *M. foliosa*. (**B**) *M. capricornis*.

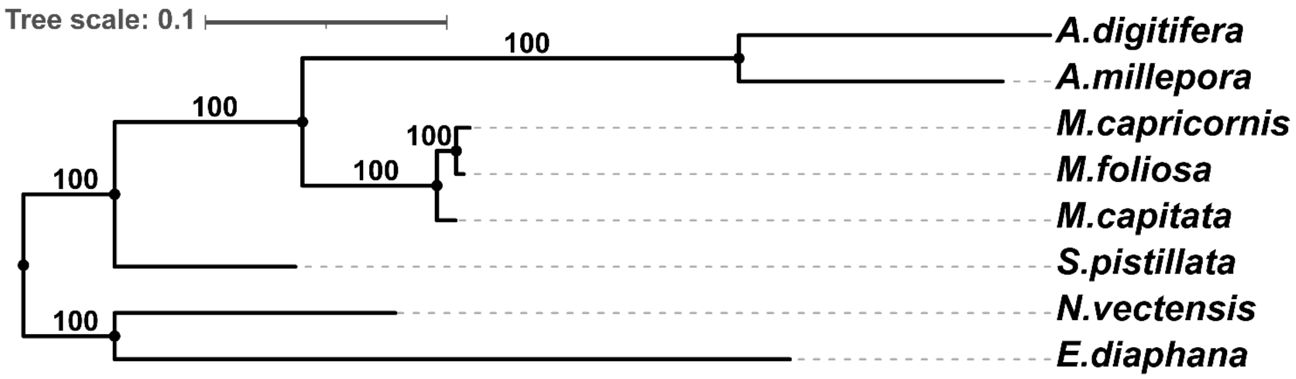

Supplementary Figure 2. Phylogenetic analysis of cnidarians constructed by using the JTT+I+G+F model with 1,000 bootstrap. The scale bar represents branch lengths. Nodes with 100% bootstrap support are indicated.

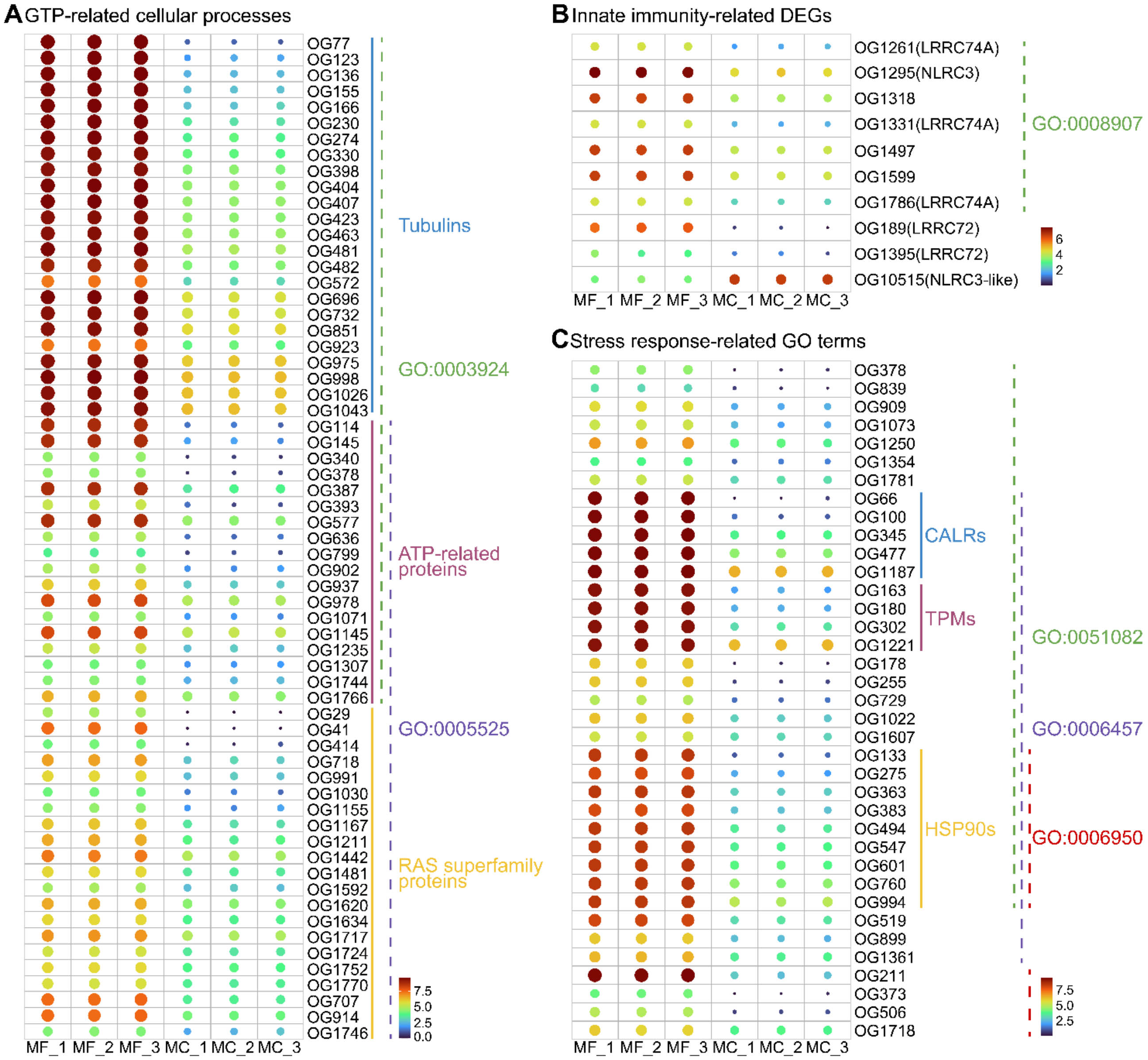

Supplementary Figure 3. Upregulated DEGs in *M. foliosa* compared to *M. capricornis* enriched in GO terms involving the interaction and regulation of GTP molecules within cells (**A**), innate immunity (**B**), and stress responses (**C**). Deep red color and bigger circles indicate genes with high expression, while deep navy blue color and smaller circles indicate genes with low expression. The number in the figure legend represents the value of log2(normalization readcount+1).

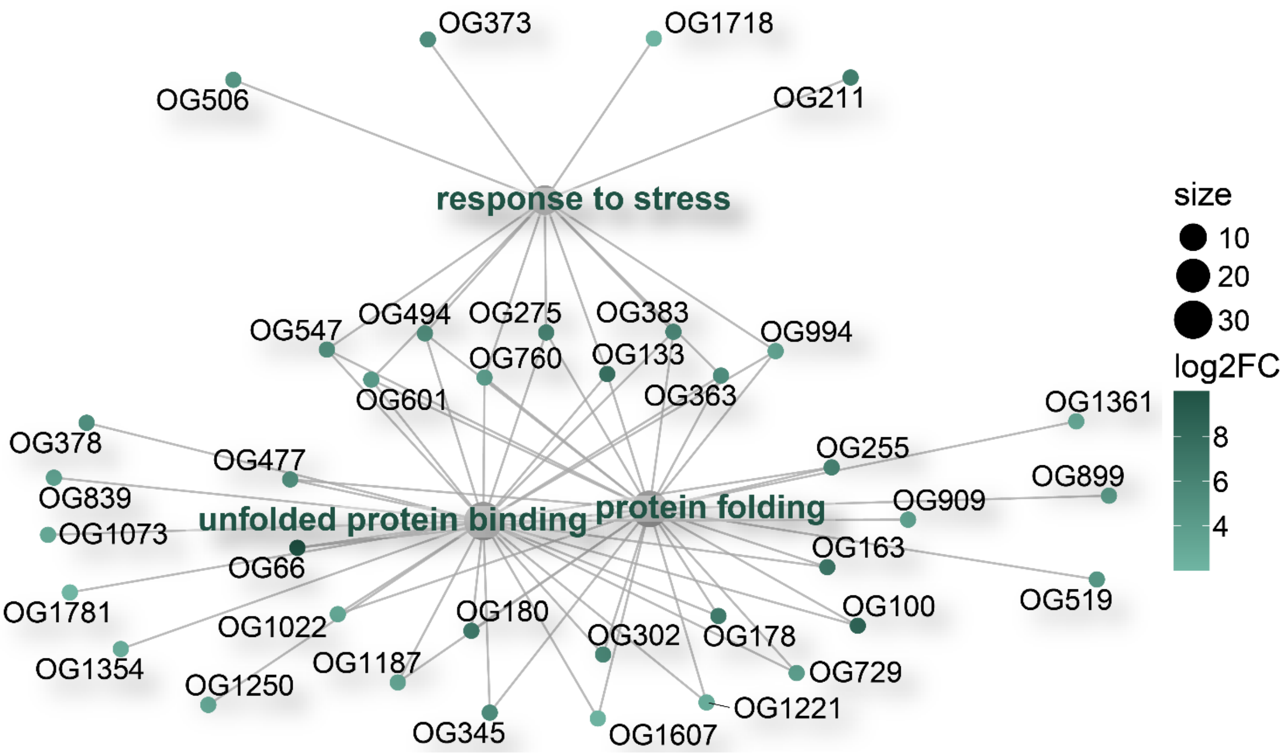

Supplementary Figure 4. The DEGs that showed significant upregulation in *M. foliosa* were enriched in GO terms related to stress response. The corresponding genes have been annotated in **Figure 6A**.

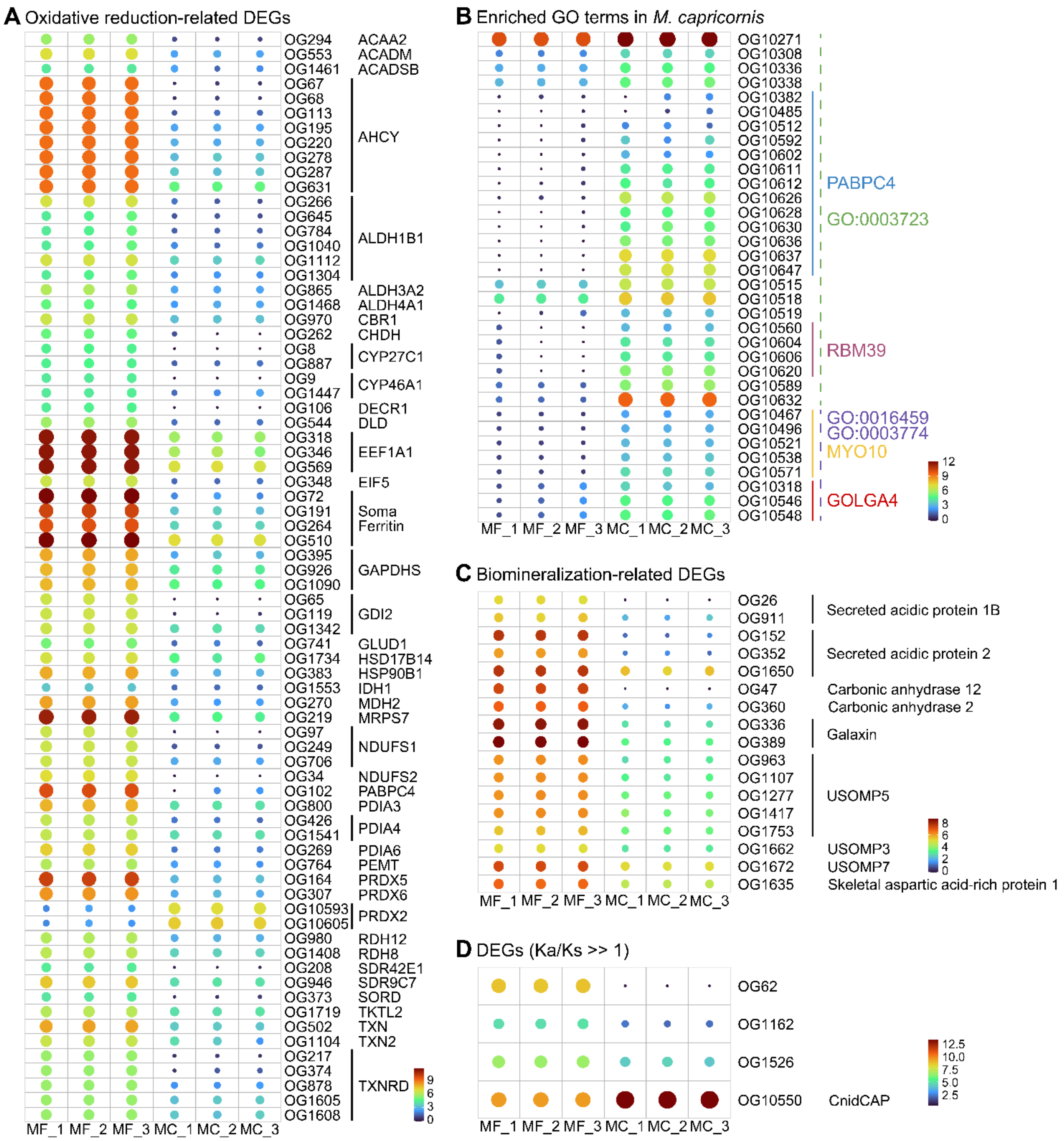

Supplementary Figure 5. (**A**) Upregulated DEGs in *M. foliosa* compared to *M. capricornis* enriched in GO terms involving oxidative-reduction processes. (**B**) Upregulated DEGs in *M. capricornis* compared to *M. foliosa* enriched in three GO terms. (**C**). Upregulated DEGs in *M. foliosa* compared to *M. capricornis* related to biomineralization processes. (**D**) DEGs with Ka/Ks ratios markedly greater than 1 in two coral species. Deep red color and bigger circles indicate genes with high expression, while deep navy blue color and smaller circles indicate genes with low expression. The number in the figure legend represents the value of log2(normalization readcount+1).

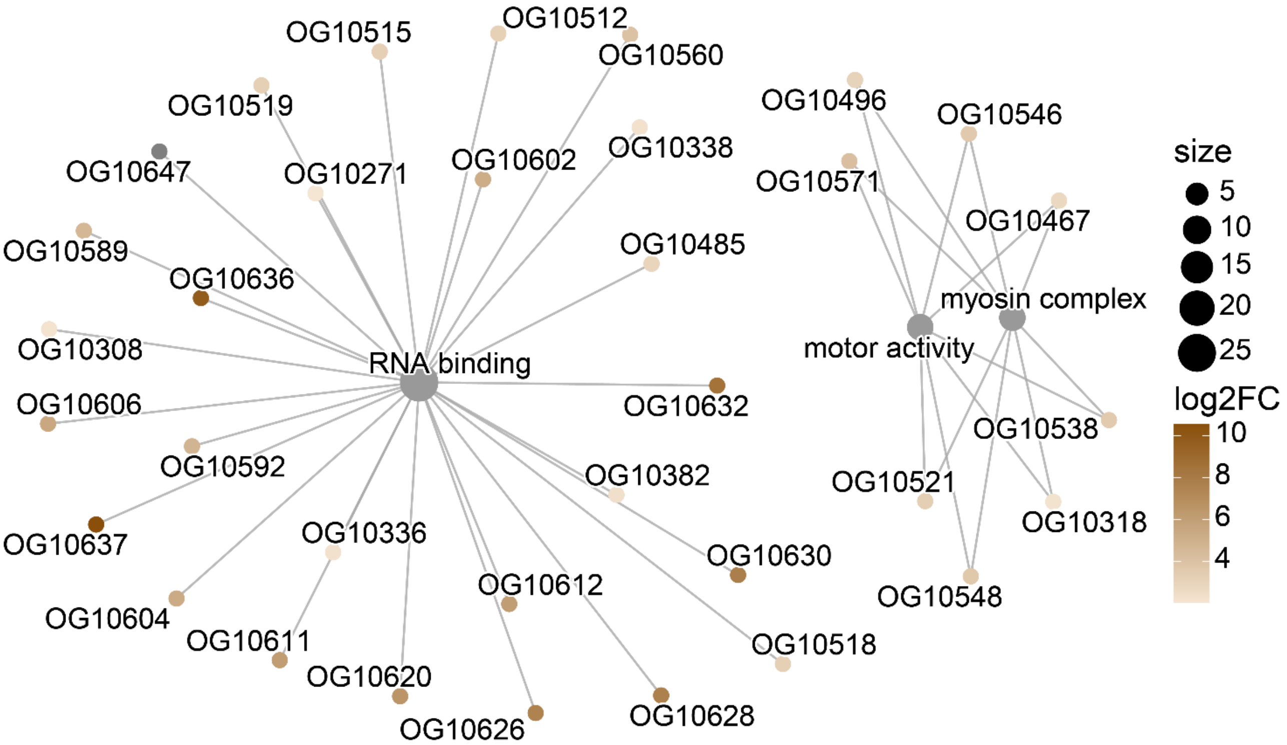

Supplementary Figure 6. GO enrichment analysis of the DEGs that showed significant upregulation in *M. capricornis*. The corresponding genes have been annotated in **Figure 6C**.
